# Using GPS tracking to monitor the breeding performance of a low-density raptor improves accuracy, and reduces long-term financial and carbon costs

**DOI:** 10.1098/rsos.221447

**Published:** 2023-08-30

**Authors:** M. Murgatroyd, G. Tate, A. Amar

**Affiliations:** ^1^ FitzPatrick Institute of African Ornithology, University of Cape Town, Rondebosch, Cape Town 7701, South Africa; ^2^ HawkWatch International, 2240 South 900 East, Salt Lake City, UT 84106, USA; ^3^ The Endangered Wildlife Trust, 27 and 28 Austin Road, Glen Austin, Midrand, Johannesburg 1685, South Africa

**Keywords:** biodiversity monitoring, carbon emissions, funding, GPS tracking, productivity, raptors

## Abstract

Traditionally, demographic monitoring of birds has been undertaken by intensive monitoring of nesting sites. However, this is challenging for low-density species, whereby the effort and costs involved in locating and monitoring remote sites can be prohibitive or even bias research findings. We show that Global Positioning System (GPS) tracking can overcome these challenges for a low-density raptor. Field monitoring of martial eagles *Polemaetus bellicosus* from 2013 to 2021 showed consistently poor breeding performance, with a mean productivity of 0.22 (±0.04) fledged young/pair/year. Using GPS tracking data to infer breeding performance gave a significantly higher productivity of 0.46 (±0.10) fledged young/pair/year. Breeding rate and success were also underestimated by field monitoring. These differences were likely due to logistical constraints of field monitoring, particularly relating to finding alternative nests. Comparing costs between approaches, we estimated that GPS monitoring was financially cheaper than field monitoring per sample after 10 years. Carbon costs per sample were lower for GPS-based approaches than field monitoring from the second year, and over a 10-year period GPS monitoring produced considerable savings (200% less carbon). We recommend that despite high initial costs, for long-term demographic monitoring of low-density species, or where logistical constraints make traditional field monitoring inaccurate, remote monitoring options should be considered.

## 1. Introduction

Comprehensive species monitoring requires demographic data to understand population dynamics, and such data can be crucial for diagnosing causes of population declines [1]. Demographic data can be used to build population viability models, which can be key to understanding population dynamics and trends. Such models are increasingly being used to inform endangered species management, for example by using them to better predict population trends under varying management scenarios. However, unreliable trends or management actions can arise when unreliable demographic data are used [2]. In the case of long-lived species that experience annual fluctuations in breeding rates, it is often necessary to conduct long-term studies to gather appropriate demographic information.

Our ability to quantify species' population changes and understand their drivers are often hampered by the resources available to monitor change at an appropriate scale and in a cost-effective manner [3]. A variety of technologies are now being widely used to overcome some of these challenges and are likely to become even more important in the future [4]. For example, acoustic recorders may offer potential opportunities to monitor species diversity and abundance in an effective manner in remote locations [3]. For some species long-term studies are required to estimate breeding parameters which may inform conservation measures [5]. However, despite technological advances, current approaches to collect these demographic parameters usually require labour intensive *in situ* fieldwork, with its associated challenges and costs.

Across the globe, large, long-lived, wide-ranging species which occur at low densities show the greatest levels of declines and highest rates of extinction [6], with these patterns apparent for both birds and mammals [7,8]. Priority research is, therefore, often directed at such species which may also function as flagship or umbrella species [9]. For birds, the monitoring of large species, such as raptors, can present specific challenges [10]. Raptor species have shown some of the largest declines among avian species, and globally at least 50% of raptor species have declining populations [11]. While migratory, colonial or other spatially grouped species can be monitored via annual counts [12–14], monitoring of non-migratory, territorial raptors is usually only effective via nest site monitoring [15] and this presents unique challenges for species which occur at low densities or within regions where lack of infrastructure (e.g. roads) may compromise monitoring efficiency [10].

Africa is an important area for raptors [9], but severe declines in many raptor populations have been recorded across the continent in recent decades [16–19]. Worryingly, many of these declines have occurred within protected areas, suggesting direct habitat loss is unlikely to be their sole driver [16,20]. The causes of many of the declines in raptor populations are not entirely clear [19]. However, in the absence of threats causing increased mortality, reduced breeding performance can lead to population declines in raptors [21–23]. Thus, long-term monitoring of a species’ breeding performance can help to understand the demographic parameter that may be driving population declines and can help inform conservation interventions [24].

Monitoring the nests of low-density species over large areas can be difficult, particularly where infrastructure for traversing the ground is limited, which is often the case in developing countries, where biodiversity is richest and where research is most limited [25,26]. Monitoring species in such habitats requires significant effort and costs, and may even require aerial surveys to find nests over large areas [27,28]. Some territorial species may also build and use alternative nests in different years [29,30], and finding these alternative nests presents another challenge to monitoring the productivity of such species. If intensive methods (e.g. territory wide searches) are not employed to identify and check all such potential nest sites this could underestimate occupancy rates and breeding productivity, because it may be incorrectly concluded that territories are vacant or that breeding has not taken place [30]. Underestimation of breeding productivity in this manner can have ramifications on predictions of population growth and the demographic drivers of declines for endangered species. Thus, for monitoring birds with low population density and large home ranges, such as large raptors, innovative solutions are needed that allow accurate demographic monitoring to be undertaken in a cost-effective manner.

Global Positioning System (GPS) tracking devices have provided unique insights into how birds use space [31,32] and have also proven useful to find nest locations based on recursive movements to the same location, allowing researchers to infer nesting behaviour and success based on re-visitation patterns [33]. This has been demonstrated with 86–100% success for lesser kestrels *Falco naumanni* and Mediterranean gulls *Ichthyaetus melanocephalus* [33]. However, high initial costs associated with GPS tracking devices may discourage using this method when establishing a monitoring programme. There are also concerns over technology divorcing biologists from a field-based understanding of animal ecology [34].

As the cost of technology, such as tracking devices, continues to decline and our ability to make meaningful biological inferences from the data improves, more studies are adopting GPS tracking as a research method to determine breeding activity [35]. Thus, it is possible that in the long term, for low-density raptors, GPS tracking could provide a more cost effective and accurate approach to estimate breeding performance than traditional field-based monitoring. Such an approach could also lead to a reduction in the financial cost and the carbon footprint of monitoring activities, which is an aspect that is receiving increased consideration [36,37].

The martial eagle *Polemaetus bellicosus* is the largest species of eagle in Africa and has a home range of around 100 km^2^ [38]. This species, like many eagle species, may have several alternative nests within its territory [30]. Martial eagles have recently been uplisted to Endangered following declines seen in multiple parts of their range. Within South Africa, their reporting rates have declined by around 60% [20,39]. Although these declines were greater outside of protected areas (64%), significant declines also occurred within protected areas. Even within South Africa's largest protected area, the Kruger National Park (KNP), 54% population declines of martial eagles have been recorded [20]. In this study, we explore if using GPS tracking devices, compared to conventional field methods, can more accurately monitor the breeding performance of this species across the KNP. We also calculate and compare the cost-effectiveness of monitoring methods, in terms of both financial and carbon costs per sample, to identify the optimum method for long-term monitoring of this low-density species.

## 2. Methods

### 2.1. Study area

**Figure 1.**
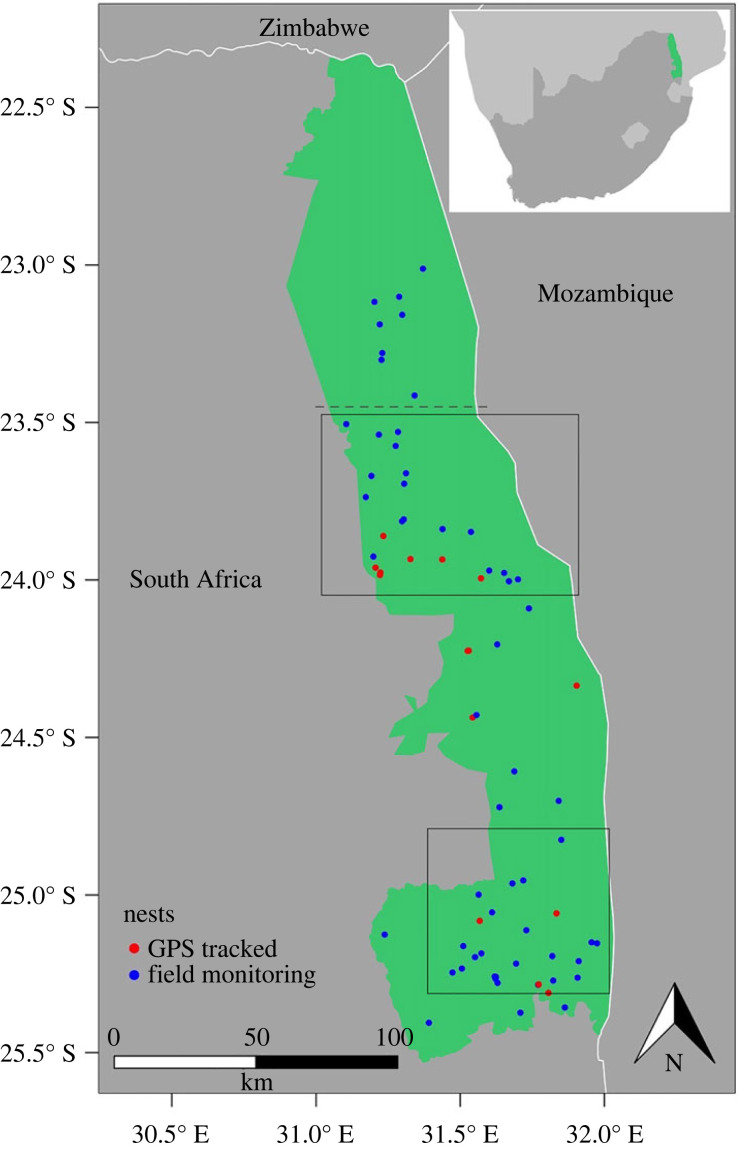
Map of Kruger National Park (green) in South Africa, showing the distribution of martial eagle nests monitored from 2013 to 2021. Nests were monitored by field monitoring (blue) and remotely by GPS tracking (red). Boxes show approximate areas covered by aerial surveys. No nests in the north of the park (above dashed line) were monitored in 2020 and 2021.

At *ca* 20 000 km^2^, the KNP is the largest protected area in South Africa and one of the largest national parks in Africa [27]. KNP lies in a semi-arid savannah biome, with rainfall mostly in the summer months from November to April [40,41], outside the main breeding season for martial eagles in this region [42]. KNP has two distinct rainfall regions, with the areas south of approximately 24.0° S (figure 1) receiving an average of 200 mm more rain per year compared to areas further north [40].

### 2.2. Estimating productivity from field monitoring

Martial eagle nests were monitored over 8 years, from 2013 to 2021. The research was approved and permitted by South African National Parks (permits: AMAA1374, AMAA1651, AMAA165). Ethical approval for fieldwork and GPS tracking (see below) was also granted by the University of Cape Town Biological Sciences Animal Ethics Committee (permits: 2013/V7/AA, 2016/v12/AA, 2019/V22/AA). Martial eagles build stick nests, which can generally be differentiated from nests of other raptors by their large size, the relative size of sticks used (i.e. although the nest structure might be similar in size to that of other raptors, e.g. African fish eagle *Haliaeetus vocifer*, the individual stick size is larger), and the location in the tree, which is usually in the highest large fork. The location of the nest in the tree means that nests are often within the leafy canopy and can be difficult to locate from long distances. This is compounded by generally thick bush and large search areas, meaning that finding all active nests within KNP is not possible. Additionally, within KNP there are vast areas that are distant from roads, making nest finding in this environment logistically challenging. Nests were located using a combination of aerial surveys, from helicopters in 2011, 2014 (north box only, figure 1) and 2015 (south box only, figure 1) [27,43] and a high-winged aircraft in 2020 (opportunistically), as well as being located while on foot or from vehicles in the study area. Other nests were reported to us by rangers or tourists.

Each year we visited as many territories as logistically possible, visiting all known nest sites in the territory to determine the breeding performance and outcome. We aimed to visit all nests at least once in the incubation window (from April to June) and for those nests where breeding attempts were recorded (i.e. an egg was laid), we aimed to visit at least twice more to determine the breeding outcome. In later years (2020–2021), nests in the northern region of KNP were not visited (figure 1) due to time constraints and because most nests in this area had completely collapsed or fallen into disrepair. Although nestlings or incubating eagles could sometimes be seen in aerial surveys, the timing and frequency of these surveys were not sufficient to assess productivity or occupancy.

During each nest visit, we examined the nest content using an action camera on a telescopic pole. We recorded presence of green vegetation lining (suggesting evidence of territory occupancy [44]), presence of an egg or nestling, or if the nest was empty. When a nestling was present, its age was estimated visually from feather growth, and photos were cross-checked against nestlings of known age [45]. We also recorded presence of adult martial eagles close to the nest, presence of moulted eagle feathers, presence of prey remains or if mutes (i.e. white faecal matter) were present below or on the nest, the presence of which indicates that the nest was likely occupied. Some nests were too high (greater than 15 m) for the pole to reach (until 2021 when a longer pole was used) and thus occupancy and activity were inferred from evidence available on the ground or the observation of adults or a nestling on the nest. Although martial eagle nestlings can take 90–120 days to fledge, failures in the late stage of nestling development are less common, and for practical reasons nests were considered successful when a large feathered nestling (greater than *ca* 56 days) was seen at the nest [43,46].

Nest were considered ‘occupied’ if the nest was lined or a breeding attempt was recorded, or if there were signs of occupancy (green lining, prey remains, moulted feathers). Mutes alone were not used to determine occupancy since they may have come from another raptor species. However, heavy muting at active nests is a good indication that a nestling had hatched, and could be used to determine activity at high nests when our camera could not reach.

Occasionally nests were usurped by other species (e.g. African hawk eagle *Aquila spilogaster*, Verreaux's eagle owl *Bubo lacteus*, white-backed vulture *Gyps africanus* and bateleur *Terathoplus ecaudatus*). When this occurred martial eagles rarely used the nest again, and thus these nests were treated as ‘unoccupied’ unless confirmed otherwise by field monitoring. When a nest was unoccupied for three or more consecutive years and there were no further signs of occupancy again for the study duration, all data after the last occupied year were removed from our analysis. We also removed nests that had only 2 years of data and that were unoccupied in both years.

Some martial eagle pairs had more than one nest within their territory. Based on previous estimates of inter-nest distances [43], nests less than 3 km apart were treated as being from the same territory, except when breeding attempts were known to occur at the same time, or when GPS tracking data confirmed which territory a nest belonged to. All measures of breeding performance (breeding rate, breeding success and productivity) were calculated on a territory (not nest) level. The breeding rate was calculated as the proportion of territories in which a breeding attempt occurred; breeding success was the proportion of attempts which successfully produced a fledgling; and the breeding productivity was the proportion of territories which successfully produced a fledgling [44].

Each year for which data were available and signs of occupancy were present was considered an ‘occupied territory year’. Martial eagles are usually biennial breeders. Therefore, any territories with only 1 year of data were removed from these analyses since a single year is unlikely to represent average breeding performance at any territory. Lastly, when calculating breeding performance based on field monitoring, we removed all territories for which we also had GPS tracking data (below), since these territories were not subject to the same biases as normal monitoring data, particularly where we had uncertainty over whether other alternative nests were present within the territory.

### 2.3. Estimating productivity from GPS tagged birds

From 2012 to 2020, 14 adult martial eagles (*n* female = 9, *n* male = 5) were caught using a bal-chatri trap baited with a domestic chicken, and fitted with GPS tags. Three types of GPS tags were used (manufactured by Microwave Telemetry© 70 g *n* = 6 and 45 g *n* = 4, and MadeByTheo© 40 g *n* = 4). Overall, tags weighed 0.9–2.1% of average adult martial eagle weights (males, 3.3 kg; and females, 4.7 kg [47]). All tags were fitted using a Teflon© back-pack style harness attached with a cotton weak-link [48]. Microwave telemetry tags transmitted their data via satellite, while MadeByTheo tags transmitted data via cell phone network. Throughout the study period all GPS data were automatically sent to MoveBank©, where they were examined visually to find clusters of locations which suggested nest locations. These nest locations were visited in the field as per the monitoring above, with the advantage that when tracked martial eagles used a new nest location this could be easily detected from the tracking data.

Additional to the monitoring described above (i.e. field monitoring guided by tracking data), we also estimated productivity for tagged birds based on tracking data alone. Due to the fact that martial eagles only lay one egg per breeding attempt, we were confident that all successful breeding attempts inferred from GPS only ever produced one fledged young, and thus productivity values could be easily calculated without having to account for multiple nestlings produced by some other species. All processing and analyses were performed in R [49]. GPS locations were recorded on an hourly basis (Microwave Telemetry© tags) or up to every 3 s (MadeByTheo© tags). Therefore, we used the ‘track_resample’ function in the ‘amt’ package to subsample points to a hourly basis (with a 5 min tolerance) [50]. Following this ‘nestR’ was used to ascertain annual breeding rate, success and productivity of tagged birds [51]. nestR uses the GPS tracking data to look for re-visitation to locations, and measures the frequency and duration of those re-visitations to determine breeding performance based on user-defined criteria on seasonality and GPS data availability (table 1). Male and female martial eagles can be differentiated by their reversed sexual dimorphism, whereby males are smaller than females. For all tagged eagles the sex was known. For some tracking attributes (percentage of days a nest site is visited, and percentage of fixes at the nest on the day with maximum attendance) we ascribed attributes differently for males and females, given that we expect females to spend more time at the nest during the breeding season, particularly while incubating and brooding young chicks, compared to males.

**Table 1.** Details of the user-defined attributes used to program ‘nestR’ to determine breeding performance of GPS tagged martial eagles.

seasonality	user-defined input	description
season start date	1 Apr	start of breeding season
season end date	11 Nov	end of breeding season
nest cycle	106 days	duration to define successful (i.e. 50 days incubation + 56 days nestling rearing)
*GPS data*		
buffer	150 m	buffer around points to measure re-visitation to allow for GPS error
Min_pts	4	number of fixes in a buffer to be retained for re-visitation calculations (when there were fewer than 4 fixes per day (mean) this was reduced to 2)
Min_d_fix	4	minimum number of fixes in a day required to count a day as ‘unvisited’
Min_consec	7	minimum number of consecutive days a location is visited to detect a breeding attempt
Min_top_att	80 (females), 35 (males)	the per cent of fixes at the location on the day with maximum attendance (when there were fewer than 8 fixes per day (mean) this was reduced to 35 for females too)
Min_days_att	40 (female), 30 (males)	the per cent of days a location is visited between the first and last visit (when there were fewer than 8 fixes per day (mean) this was also reduced to 30 for females)

For all breeding years, where GPS data were available for some or all of the breeding season, we ran nestR to identify nests and to get a probability of breeding outcomes using a ‘phi_time_p_time’ model in which detection probability decreases with time and survival varies through time. At one territory for 1 year both the male and female eagles were tracked. To avoid replicates we only used the female eagle's GPS data for this nest, although visual checks were performed on the male's GPS data.

### 2.4. Comparing breeding performance using different monitoring methods

We estimated and compared breeding performance using three methods: (i) with only field data (excluding GPS tracked birds), (ii) with only GPS data and (iii) using a combination of both methods for the GPS tracked birds (i.e. field-corrected GPS data). For each of these methods we aimed to calculate the breeding rate, the breeding success and the breeding productivity.

For our analyses of field data, if nests were not checked during the incubation window, or if the occurrence of a breeding attempt could not be confirmed (e.g. when the pole did not reach and the visit was inconclusive), then those breeding years were not included in analyses of breeding rate or breeding success, but could be used for breeding productivity if conclusive data on breeding outcome were collected later in the year. As such, all nests where it could be determined if a nestling was reared successfully or not, regardless of when the nest was first monitored, were used to calculate the annual breeding productivity of occupied territories. Territories where at least one of the adult birds was also GPS tracked were not included in this analysis, so that this scenario represents using field monitoring only.

When using GPS data to determine breeding performance, we only used the years when sufficient GPS data were collected to determine breeding performance (i.e. full coverage from March to November, and more than an average of two fixes per day). We did not supplement the outcomes of nestR with any field findings, so that this analysis would represent a ‘remote monitoring’ scenario if no fieldwork was carried out.

Lastly, when using both methods to determine breeding performance, for years when the results of nestR contradicted our findings in the field, we gave preference to the results that were confirmed in the field. Thus, this ‘field-corrected’ scenario represented using remote monitoring to ensure no nesting attempts were missed, but using field monitoring to confirm the outcome of those attempts, and may be considered the most accurate method.

To explore whether estimates of each breeding performance measure differed between the methods used, we built a binomial GLMM for each breeding parameter [52]. The models included the fixed effect ‘method’, and a random term for ‘territory’ and ‘year’ to account for the fact that we had repeated observations from the same territory and data collected across different years. The response variable was the breeding parameter (i.e. breeding rate, success and productivity). We then used ‘emmeans’ to look for pairwise differences between the methods [53].

### 2.5. Financial and carbon costs of different monitoring methods

We assessed the basic financial costs and the carbon emissions associated with monitoring using fieldwork methods or monitoring using purely remote tracking, as well as a hybrid method which combines both approaches by using GPS tracking to monitor eagles over most of the year and visiting the field site once per year to confirm breeding productivity. Each of these was calculated over a 10-year period. For fieldwork, we considered the average daily cost of accommodation, transport, field stipends and wages for two people, and assumed six weeks of work per year across three trips to achieve all fieldwork. Additionally, for fieldwork we included the costs of four aerial surveys which were undertaken to locate nests using either helicopter (80 h) or fixed wing aircraft (40 h). All these aspects are based on our own experience of effort required to monitor this population. For GPS tracking, in the first year we calculated the cost of 10 transmitters manufactured by Microwave Telemetry©, the most frequently used tag in this study and included a single one-off cost of 60 days of fieldwork (one trip) to deploy tags, using the same average daily fieldwork rates (electronic supplementary material, table S1). To maintain at least 10 tracked eagles per year, we included the cost of one new tag and one refurbished tag (assuming one tag recovery) biennially, and 10 days of fieldwork to recover any potentially ‘downed’ (fallen off, or mortality event) tags and to deploy these tags. Each year we presumed the annual data transfer costs for 10 active tags, and the cost of a data analyst for 3 days per year to check and analyse breeding performance. As a hybrid approach, we assumed all of the costs associated with GPS tracking, and added the costs of one trip per year to confirm the breeding productivity assumed by GPS tracking. All financial costs are reported in US dollars ($) (but see electronic supplementary material, table S1, for values in local currency) and we assumed a 6% annual increase in costs due to inflation. To calculate the carbon footprint, we considered travel to and from KNP (flights), mileage within KNP using a 4 × 4 vehicle, the manufacture of tags (based on the most similar estimates for mobile phones) and the transmission of data. For the three monitoring methods we calculated the annual costs per sample; for fieldwork we assumed the monitoring of 17 territories per year (the average previously monitored for breeding productivity; electronic supplementary material, table S2), and for GPS and hybrid methods we assumed a sample size of 10 tracked eagles per year.

## 3. Results

### 3.1. Breeding performance estimates from field monitoring

**Figure 2.**
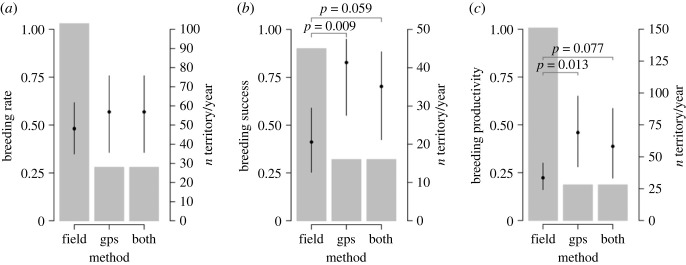
Comparisons of three measures of martial eagle breeding performance recorded by three methods (i.e. ‘field’ = field monitoring, ‘gps’ = using GPS tracking data to infer breeding performance, ‘both’ = corrected GPS tracking data). Dots ±95% confidence intervals show (*a*) breeding rate (the proportion of territories where an attempt was recorded), (*b*) breeding success (proportion of attempts which were successful) and (*c*) breeding productivity (proportion of monitored territories which were successful). Bars (*z*-axis) show number of territory years in each group for each analysis. *P*-values indicating differences in breeding performance between methods are given where they are less than 0.1; no other between-group differences approached significance.

We undertook field monitoring for 157 ‘occupied territory years’ across 39 unique territories of untagged martial eagles (see electronic supplementary material, table S2, for annual sample sizes). Within these 39 territories, only 23% (*n* = 9) had more than one nest of which we were aware, with a maximum of four nests at one territory. Nest usurpation occurred in around 6% of territory years. We had reliable information on breeding attempts from 103 of the 157 years of monitoring, and from these 103 territory years there were 50 breeding attempts, giving a breeding rate estimate (±s.e.) of 0.48 (±0.07) attempts per pair (table 2, figure 2). This included eight instances of breeding in two consecutive years, of which two were two consecutive successful years, and one instance of three consecutive attempts; however, only one was successful (6% of attempts occurred in consecutive years). The outcomes of 45 attempts were known, with a breeding success estimate of 0.41 (±0.09) young fledged per attempt (table 2, figure 2). Whether a territory was successful or not was known for 151 occupied breeding years, giving a breeding productivity estimate of 0.22 (±0.04) fledged young per pair per year (table 2, figure 2).

**Table 2.** Model estimates and standard error (s.e.) of martial eagle breeding performance and sample sizes (*n* years = total number of territory years; *n* attempts = total number of breeding attempts recorded; *n* successes = total number of chicks fledged) of each method.

method	breeding rate				breeding success				breeding productivity			
	estimate	s.e.	*n* years	*n* attempts	estimate	s.e.	*n* years	*n* successes	estimate	s.e.	*n* years	*n* successes
field monitoring	0.48	0.07	103	50	0.41	0.09	45	18	0.22	0.04	151	34
GPS	0.57	0.11	28	16	0.83	0.10	16	13	0.46	0.10	28	13
both	0.57	0.11	28	16	0.70	0.12	16	11	0.39	0.10	28	11

### 3.2. Breeding performance from GPS tags alone

**Figure 3.**
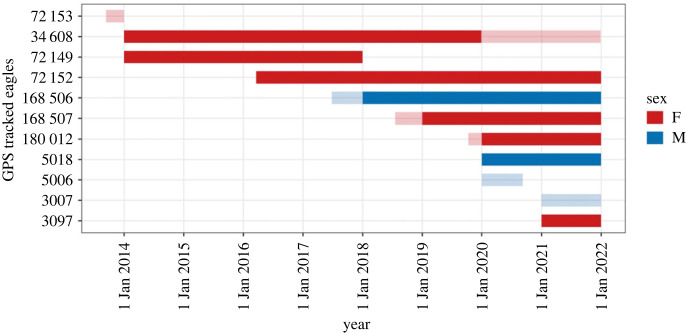
Tracking data for adult martial eagles which were found to be territory holding. Transparent bars are data excluded from our analyses either due to insufficient annual tracking duration (e.g. eagle 180 012 during 2018), too few average points per day for the given year (e.g. eagle 34 608 during 2020 and 2021), or to avoid pseudo replication when the tracked eagles were part of the same pair (e.g. eagle 3007 is the mate of eagle 3097).

Of the 14 GPS tagged adult eagles, 11 were territory holding and came from 10 different breeding pairs or territories, and eight had adequate data to assess breeding performance in at least 1 year (figure 3). From these eight martial eagles, we had 28 ‘occupied territory years’ (figure 3). Based on GPS tracking data, we identified that 50% had more than one nest, with a maximum of three nests for one individual. Across the 28 observations, nestR identified 16 breeding attempts, giving an average breeding rate of 0.57 (±0.11) attempts per year (figure 2). This included four instances where individuals bred in two consecutive years (14% of attempts occurred in consecutive years). Three of these were successful attempts following a failed year, while one was an unsuccessful attempt following a successful year. The outcomes of 16 breeding attempts were estimated by nestR, with a mean success of 0.83 (±0.10) fledged young per attempt. The mean breeding productivity of GPS tracked eagles as estimated by nestR was 0.46 (±0.10) fledged young per pair per annum (figure 2).

### 3.3. Breeding performance from field-corrected GPS data

We then used the breeding data from the same GPS tracked individuals but corrected when field monitoring showed a different outcome. Field-corrected GPS data suggested a breeding rate of 0.57 (±0.11) attempts per pair per year. Breeding success, based on these 16 attempts, was 0.70 (±0.12) fledged young per attempt. Overall, using field-corrected GPS data the outcome of 28 breeding observations gave a mean productivity of 0.39 (±0.10) fledged young per pair (table 2, figure 2).

### 3.4. Comparison of estimates between the different methods

For breeding rate there were no significant differences recorded between the monitoring methods, although the use of GPS data resulted in higher estimates of breeding rate than field monitoring alone. Using GPS data alone or field-corrected GPS data resulted in the same estimates of breeding rate and these were biologically meaningful, in that the breeding rate was shown to be greater than 0.5 attempts per pair per year (figure 2*a*).

Breeding success and productivity were significantly lower for birds based on field monitoring estimates compared to using GPS data (success: *X* ^2^= 1.93, d.f. = 2, *p* = 0.009; productivity: *X*^2^ = −1.09, d.f. = 2, *p* = 0.013; figure 2*b*,*c*). Breeding success and productivity were also lower for birds based on field monitoring estimates compared to using field-corrected GPS data, although these differences were marginally non-significant (success: *X*^2^ = 1.22, d.f. = 2, *p* = 0.059; productivity: *X*^2^ = 0.79, d.f. = 2, *p* = 0.077; figure 2*b*,*c*).

Field-corrected GPS data suggested slightly lower success and productivity rates compared to GPS data only (success rate: 0.70 versus 0.83; productivity rate: 0.39 versus 0.46). The lower success rate using combined data sources was due to two occasions when eagles undertook an extended incubation of addled eggs. GPS data alone suggested that these were successful breeding attempts, while breeding failures were confirmed by fieldwork. However, there were no significant differences between any of the estimates of breeding performance recorded by these two methods (figure 2).

### 3.5. Estimated financial and carbon costs per sample of field versus GPS monitoring

**Figure 4.**
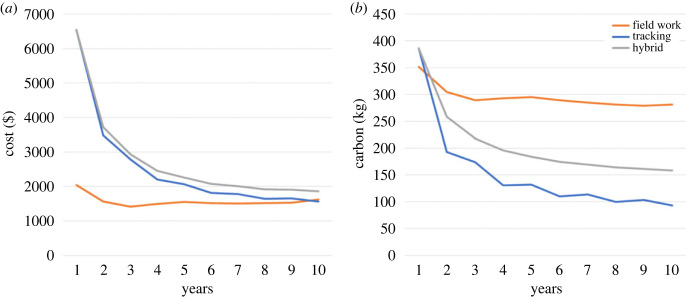
Cost analysis showing the annual cost per sample for (*a*) the financial cost and (*b*) the carbon cost of monitoring martial eagle breeding performance using either fieldwork or remote monitoring via tracking study, or a hybrid approach. See electronic supplementary material, table S1, for full costs breakdown and a reproducible framework for calculating relative costs.

In the first year, we estimated that cost per sample (territory per year monitored) with a project using GPS tags was around 220% higher than taking a purely field monitoring approach ($6539 versus $2039). However, after just 2 years this difference had nearly halved ($3487 versus $1559), and over the long term the cost per sample of GPS tracking was comparable to, or even slightly cheaper than, field monitoring (figure 4*a*). Financially, the hybrid approach was also substantially more expensive than field monitoring alone in the first year; however, after a 10-year period this approach only cost around 15% per sample more than field monitoring alone ($1857 versus $1620) (figure 4*a*). Carbon costs per sample associated with a GPS tracking or a hybrid approach were only slightly higher than a purely field monitoring approach in the first year (figure 4*b*). However, over a 10-year period carbon costs per sample in a fieldwork monitoring programme became around 200% greater than using GPS tags alone (281 versus 93 kg per sample).

## 4. Discussion

Our results suggest that the methods chosen to monitor breeding martial eagles over a large area can have a considerable impact on estimates of breeding performance. Based on our comparisons, using field methods alone to monitor this population is likely to underestimate productivity by over 70% (breeding productivity: 0.22 using field data versus 0.39 using field-corrected GPS data), whereas using GPS data alone may overestimate productivity by around 18% (breeding productivity: 0.46 using GPS data versus 0.39 using field-corrected GPS data). Additionally, our cost analysis suggests that despite higher initial financial costs of monitoring using GPS tags, this method can become cost effective and over a 10-year period would be around the same cost per sample, or even slightly cheaper than field monitoring. Furthermore, carbon emission savings by using GPS monitoring rather than traditional field monitoring start to pay off in the second year and can result in 200% less carbon emissions per sample in the long term. A hybrid approach could offer the best approach in the long term, by only costing around 15% more per sample financially than frequent field visits, while emitting 70% less carbon emissions per sample and providing the most reliable breeding estimates.

GPS tracking data suggested that using field monitoring alone greatly underestimates the number of alternative nests. Using field methods alone, we found only around 23% of territories had alternative nests, whereas across our GPS tagged birds around 50% of territories had alternative nests. Assuming that our relatively small sample size of GPS tagged birds is representative of the population, this suggests that we are missing many of the alternative nests of the pairs that we have been monitoring and this is likely to cause an underestimation of the breeding rate. To a degree we may have corrected for this by excluding territories from our analysis when the monitored nest was not used for 3 years or more. This correction attempted to remove territories from the breeding rate estimate where eagles may have switched to using an unknown alternative nest, and this is likely reflected by the fact the differences in breeding rate between then methods were non-significant. Nevertheless, the breeding rate at territories monitored using GPS tracking was 21% higher than those monitored using only field observations (0.57 versus 0.47). This potential bias has been highlighted by previous studies of large eagles which use alternative nests and stresses the importance of ensuring that all nests in a territory are located [30,54].

Our estimate of a breeding rate using GPS tracking of greater than 0.5 indicates that more than half of the population breed each year on average. It was previously assumed that within this population, as in many other populations, martial eagles only breed biennially [43] due to the long (greater than eight month) post-fledging dependency period of young birds, which inhibits any breeding attempt following a successful year [46]. Thus, underestimations of breeding rate recorded via field observations in the earlier years of this study may also be due to nests not being checked adequately in the year following successful breeding because assumptions were made that they would not be active. The study of Hustler & Howells [46] of martial eagles in Hwange National Park, Zimbabwe, suggested that while some pairs did attempt to breed in consecutive years, these were mainly pairs that had failed in the previous year. The overall breeding rate was less than 0.5 (0.46 attempts per pair); however, the annual rate appeared to vary with rainfall. Similar to our study (14%, 4 out of 28 attempts of GPS tagged birds), around 15% of birds in their study bred in consecutive years, although only two successful attempts followed successful years. Herholdt & Kemp [55] found that martial eagle breeding rate within the Kgalagadi National Park (formerly the Kalahari Gemsbok National Park) was around 0.6, and they suggest that the majority of pairs attempted to breed in consecutive years (72%, 38 out of 53 attempts). These differences suggest that frequency of consecutive breeding can vary dramatically between populations.

Breeding success was significantly lower based on field observations (0.43) compared to GPS monitoring (0.82), which also ultimately contributed to an underestimation of overall breeding productivity. Breeding success estimated from field monitoring might have been underestimated because gaps in monitoring events were too long, and thus hatching was not confirmed and fledged young were not always located. Furthermore, we should consider other biases between monitoring techniques; for nest monitoring, young were considered fledged at 56 days old, but in some cases the nest may not have been visited until later than this date. Therefore, any failures which occurred between 56 days and the next visit would be considered as ‘unsuccessful’. However, the same nest monitored by GPS tracking would have been considered ‘successful’. When more frequent monitoring is not possible, this gap could be filled in part by installation of nest cameras, which can also provide detailed information on the proximate causes of nest failure [56]. However, disturbance effects need to minimized, potentially by installing cameras prior to the start of the breeding season [57]. There are also concerns that frequent field monitoring can cause disturbance at nest sites, and particularly visits early in the breeding season may cause breeding failures [58]. Such concerns around disturbance also provide additional support for the use of remote monitoring of breeding via GPS tracking.

GPS monitoring in isolation from field monitoring led to some apparent overestimates in breeding success. In two cases, pairs incubated non-viable eggs for an extended period of time until they were eventually abandoned or predated. Re-visitation rates to these nests analysed by nestR suggested these nests were successful (because the time on the nest was in excess of the time usually required for successful breeding). Thus, it was only through additional field monitoring that these actual outcomes were known. It may be possible to examine the differences in movement between the incubation and nestling-rearing periods in more detail to provide a better tool for monitoring in the future, particularly where higher resolution tracking data are available. Nevertheless, GPS monitoring still results in estimates of breeding which are more accurate (i.e. the field-corrected GPS data) than field monitoring alone.

There have been concerns that fitting GPS devices to raptors might cause reduced fitness or breeding productivity [59,60]. Our results add support to a lack of negative impacts of GPS tracking on martial eagles, with tagged birds breeding successfully on multiple occasions. This is further supported by a recent study on a population of martial eagles in the central Karoo, South Africa. Here no significant differences were recorded in the breeding performance (productivity and breeding success) between GPS tagged (14 GPS tracked adults from 14 different pairs) versus untagged birds (18 monitored pairs) over a 3-year period [61]. Indeed, using GPS tracking to ensure that all breeding attempts are known improves estimates of breeding performance to such an extent that it would likely alter the trajectory of population modelling. Previous population modelling of martial eagles in KNP concluded that in order to maintain a stable population, productivity would need to be 0.37 young per pair per year [43]. The productivity rate we recorded from nest monitoring alone (0.22 young per pair per year) would predict a declining population, while the productivity rate we recorded from GPS tracking combined with field monitoring (0.39 young per pair per year) would predict a stable population. This has important implications for conservation management of the species. GPS tracking also has the added benefit of enabling accurate estimates of adult survival, which can further improve the accuracy of population modelling [62]. In many cases, GPS tracking can also provide information on causes of mortality, mortality rates and habitat preferences which are critical to understand when implementing conservation measures [38,63]. In maximizing the potential benefit of data, GPS data can also provide public outreach opportunities, whereby they can provide innovative tools for science engagement and communication [64,65].

Throughout our study different methods were used to locate nests (i.e. helicopter, plane, road surveys, reports from rangers and tourists), and this may have impacted the breeding performance recorded by field data. However, it is clear from our results that these field methods alone have not been sufficient to locate all alternative nests for martial eagles. It appears that although aerial survey methods are likely to locate a larger proportion of nests, only GPS tracking of individual eagles ensures that all nests are found.

It is recognized that long-term biodiversity studies are often limited by the availability of sufficient funding [66]. We show the usefulness of cost analyses of monitoring programmes beyond just the first few years, which may help to secure the long-term future of many programmes. Despite relatively large initial investment costs, the financial cost per sample of using GPS tracking can be comparable to field monitoring for long-term monitoring of martial eagles in KNP. Importantly, large savings are also seen in reduced carbon emission costs. The financial and carbon costs we have shown are specific to this monitoring programme; however, we have also provided a framework for other researchers (electronic supplementary material, table S1) to consider and calculate the costs associated with their work. These comparisons are important considering the often-limited resources available for conservation and research programmes, and the desire to minimize the negative ecological impact of research. As is the case with all sectors of society, it is becoming increasingly important to consider the carbon costs associated with biodiversity monitoring [36]. Although the exact carbon costs of our tracking units were not available, we used the closest available figure for a smart mobile phone. Despite uncertainties in the actual values of carbon emissions for this work, the substantial differences suggest that the potentially large carbon costs of fieldwork should be considered when choosing appropriate sustainable methods.

We recognize that a tracking approach may not be viable for all raptor species. For example, some species may be too difficult to trap in sufficient numbers to allow monitoring to be implemented in this manner. Other species may be too small to carry tracking devices which provide data at a sufficient resolution over multiple years (e.g. pygmy falcon *Polihierax semitorquatus*). Our financial analysis may also not apply to all systems; for example, the analysis may be different when the species occupy areas with better infrastructure or more open habitats, or where nests are easier to find. For example, this may apply to martial eagles which nest in the Karoo on powerlines [67]. The costs of the two approaches may also be very different for colonial species (e.g. colonial vultures) versus territorial species, where colonial species may allow productivity to be assessed for multiple individuals from repeated visits to the same locations (e.g. [68]).

As we expect that the costs of tracking equipment will continue to fall as a competitive market of suppliers is emerging, more researchers are likely to adopt GPS tracking as a primary method of data collection. One potential concern associated with this is that a technology-based approach may divorce biologists from a field-based understanding of animal ecology [34]. In our case, fieldwork also provides an important connection between biologists and conservation practitioners on the ground, as well as other project stakeholders (e.g. park rangers, guides and tourists). Therefore, we would recommend regular engagement sessions and outreach to provide relevant information to park staff and the public, which also helps to increase reporting rates of marked eagles and nest locations.

Our study shows the potential contribution that GPS tracking can make to overcoming monitoring biases for wide-ranging cryptic breeding raptors and especially those with multiple alternative nests [30]. The initial costs of GPS tags are often deemed to be prohibitive of long-term monitoring studies [69]. However, our cost analysis suggests that for long-term research projects, monitoring using GPS tags can become comparable to or even cheaper than field monitoring, principally because of the ongoing staff and fuel costs associated with field monitoring. Based on these results we would encourage researchers embarking on long-term breeding monitoring studies to consider using GPS tracking devices rather than field studies, and where possible to consider financial budgets beyond the first years of any study.

Importantly our study has shown that GPS technology can increase the accuracy of monitoring of the breeding productivity of a low-density large raptor, and these estimates suggest very different likely population growth for the population. While field logistics and safety in some protected areas are becoming more challenging due to the rise of poaching, it is promising that remote monitoring can yield useful information in an effective and efficient manner.

## Data Availability

Data related to financial and carbon costs are available in electronic supplementary material, table S1 [70]. Data on martial eagle breeding performance recorded by field methods are available on ZivaHub: https://figshare.com/s/3a3d3ba16e08c342c3fe (doi:10.25375/uct.21542550) [71]. Data on martial eagle breeding performance recorded by GPS tracking data, with comparative results for field monitoring only and field-corrected data are available on ZivaHub: https://figshare.com/s/705c2dfc23a23e0c21bb (doi:10.25375/uct.21542637) [72].
